# Prognostic Value of Procalcitonin in Febrile People Living with Human Immunodeficiency Virus (PLWH) Admitted to the Emergency Department

**DOI:** 10.3390/medicina61020240

**Published:** 2025-01-29

**Authors:** Luigi Celani, Luigi Carbone, Francesco Ceppa, Andrea Piccioni, Davide Antonio Della Polla, Marta Chiuchiarelli, Antonella Cingolani, Giuseppe De Matteis, Rita Murri, Antonio Gasbarrini, Francesco Franceschi, Marcello Covino

**Affiliations:** 1 Emergency Department, Fondazione Policlinico Universitario A. Gemelli, IRCCS, 00168 Rome, Italy; luigi.celani@policlinicogemelli.it (L.C.); andrea.piccioni@policlinicogemelli.it (A.P.); davideantonio.dellapolla@policlinicogemelli.it (D.A.D.P.); francesco.franceschi@policlinicogemelli.it (F.F.); marcello.covino@policlinicogemelli.it (M.C.); 2 Internal Medicine and Emergency Department, Ospedale Fatebenefratelli Isola Tiberina, Gemelli-Isola, 00168 Rome, Italy; luigi.carbone@policlinicogemelli.it; 3 Department of Infectious Diseases, Fondazione Policlinico Universitario A. Gemelli, IRCCS, 00168 Rome, Italy; marta.chiuchiarelli@guest.policlinicogemelli.it (M.C.); antonella.cingolani@policlinicogemelli.it (A.C.); rita.murri@policlinicogemelli.it (R.M.); 4 Department of Internal Medicina and Gastroenterology, Fondazione Policlinico Universitario A. Gemelli, IRCCS, 00168 Rome, Italy; giuseppe.dematteis@policlinicogemelli.it (G.D.M.); antonio.gasbarrini@policlinicogemelli.it (A.G.)

**Keywords:** HIV, procalcitonin, viremia, mortality, antiretroviral therapy, emergency department, prognostic marker

## Abstract

*Background and Objectives*: The management of HIV patients presenting with fever in the Emergency Department (ED) remains a challenging clinical scenario. Accurate risk stratification and prognostic indicators are crucial for timely intervention and improved patient outcomes. Procalcitonin (PCT) has emerged as a promising biomarker for assessing the severity and prognosis of various infectious diseases. The study aimed to evaluate the prognostic value of procalcitonin (PCT) in HIV patients admitted to the Emergency Department for clinical suspicion of infection and assess its association with in-hospital mortality. *Methods*: A retrospective analysis was conducted on febrile HIV-positive patients admitted to the Emergency Department. Clinical data were collected from 2018 to 2022. Patients were categorized based on PCT levels (>0.5 ng/dL), clinical findings, comorbidities, and viro-immunological status. *Results*: We investigated data from 289 HIV-positive patients (74% male). The median age of the sample was 54 years [IQR: 42–62], 100 (35%) patients presented detectable viremia, and the median value of CD4+ T lymphocytes was 358 [IQR: 104–531]. Elevated PCT levels (≥0.5 ng/dL) were detected in 69 (23.8%) patients. A significant association was observed between elevated PCT and increased mortality risk (*p* < 0.05). The mortality rate among patients with detectable HIV viremia was higher compared to those with undetectable viremia (*p* = 0.02). Moreover, deceased patients had statistically lower CD4+ values compared to survivors (61 [IQR: 14–186] vs. 370 [IQR: 136–548], *p* < 0.001). *Conclusions*: In febrile HIV patients admitted to the Emergency Department, elevated procalcitonin levels, low CD4+, and detectable viremia are associated with an increased risk of in-hospital mortality.

## 1. Introduction

Human immunodeficiency virus (HIV) infection remains a significant global health concern, with almost 40 million individuals living with HIV worldwide [1]. HIV-infected individuals are particularly susceptible to a wide range of opportunistic infections, and fever is a common presenting symptom that often prompts Emergency Department (ED) admission. HIV-infected people face a greater risk of developing infections due to their weakened immune system [2,3,4]. Identifying the cause of fever in this group is crucial for proper treatment. Even in the antiretroviral therapy era, respiratory tract infections such as tuberculosis and pneumonia remain more common among this population, despite CD4+ cell count restoration [5]. Community Acquired Pneumonia (CAP) is still one of the main reasons for hospitalization in PLWH [6], and fever and cough are present in almost all patients [7]. The challenge faced by emergency physicians lies in accurately assessing the severity and prognosis of fever in these individuals to guide appropriate management decisions [8]. In recent years, procalcitonin (PCT) has emerged as a promising biomarker for the diagnosis and prognosis of bacterial infections [9,10,11]. PCT, the precursor molecule of the hormone calcitonin, is synthesized and released by various tissues in response to bacterial infections and sepsis. Elevated PCT levels have been shown to correlate with the presence and severity of bacterial infections in various clinical settings [12,13,14]. While the diagnostic utility of PCT in HIV-infected patients with fever has been investigated in previous studies, there are small amounts of data regarding its prognostic value in the ED population [15,16]. Understanding the prognostic implications of PCT in HIV-infected individuals presenting with fever in the ED could have significant implications for clinical decision-making, including the appropriate initiation of empirical antibiotic therapy, the need for and the duration of hospitalization, and the general allocation of healthcare resources. The study aimed to evaluate the prognostic value of procalcitonin (PCT) in HIV patients admitted to the ED for clinical suspicion of infection and assess its association with mortality. In particular, we analyzed its relationship with comorbidities, risk of infections, and the viro-immunological status.

## 2. Materials and Methods

This is a retrospective study conducted in an academic medical center, located in central Italy, with an average attendance at the ED of about 75,000 patients annually (more than 87% adults). Based on electronic health records, we identified all consecutive PLWH admitted to the ED for fever and then hospitalized for 5 years, between 1 January 2018 and 31 December 2022. We included in the analysis all PLWH with fever at presentation to the ED or who reported fever within 24 h before ED access, and the availability of a PCT determination obtained < 24 h since ED access. We excluded from our cohort patients aged < 18 years old and pregnant women.

### 2.1. Patient Characteristics and Clinical History

All demographical and clinical variables were collected from hospital-based electronic health records. For each patient included in the study we recorded the demographical characteristics (age and sex), the fever-associated symptoms presented at ED admission (dyspnea, diarrhea, vomiting, abdominal pain, chest pain, asthenia), the viro-immunological status (HIV-RNA and CD4+ T lymphocytes), whether they were taking antiretroviral therapy or were newly diagnosed. Computerized clinical data were analyzed to obtain information regarding the patient’s comorbidities based on their past medical history and the hospital discharge diagnosis. The comorbidities were used to assess the Charlson comorbidity index [17] for each patient.

### 2.2. Laboratory Sampling and Group Definition

All patients had a blood sampling for routine laboratory testing and PCT value determination. Blood cultures were obtained based on the emergency physician’s judgment. A PCT sample was collected at the admission visit to all PLWH. The cutoff value of the PCT serum level predictive of sepsis was set at 0.5 ng/mL. Procalcitonin determination was available 24 h a day. To describe the study population, all patients were categorized according to the values of PCT. Moreover, according to the data and the final diagnosis reported on the digital files, we categorized the study population into two groups, with or without infection, defined as the CDC/NHSN Surveillance Definitions for Specific Types of Infections document.

The viro-immunological status was evaluated by the HIV-RNA value and + T lymphocyte count obtained during the hospitalization or the last available data from electronic health records before the ED admission.

Patients received empirical antibiotic therapy according to current guidelines. For patients with uncertain diagnoses, the antibiotic therapy was evaluated case-by-case by an emergency physician based on clinical judgment and laboratory findings. Local protocols for empirical therapy remained stable during the study period. None of the patients seen in the emergency department had started antibiotics in the previous 24 h.

### 2.3. Outcome Measures

The primary endpoint of the study was the all-cause in-hospital mortality. As a secondary endpoint, we evaluated the relationship between PCT levels and the diagnosis of infections in PLWH admitted to the ED, with a focus on bloodstream infections (BSIs).

### 2.4. Statistical Analysis

Categorical variables are presented as absolute numbers and percentages; continuous variables are presented as medians (interquartile ranges). Categorical variables were statistically compared by the Chi-square test or Fisher exact test as appropriate. Continuous variables were compared by the Mann–Whitney U test. Survival analyses were carried out by the Kaplan–Meier method. Significant variables at the univariate analysis were entered into a Multivariate Cox regression model, to identify the independent predictors of death risk. The results of the Multivariate Cox regression analysis were presented as the Hazard Ratio (HR) and 95% confidence interval. A receiver operating characteristic (ROC) analysis was used to determine the overall discrimination value of PCT levels for the study endpoints. The ROC analysis results were reported as the ROC Area Under Curve [95% confidence interval].

All the analyses were two-sided with a significance value set at 0.05. No a priori sample size calculation was performed because all eligible records were used in this retrospective study. The statistical analysis was made by SPSS v25 (IBM, Armonk, NY, USA).

## 3. Results

In the study period, 289 HIV-positive patients were admitted to our ED for fever. Among these, 69 patients had elevated PCT levels. We categorized patients for elevated PCT levels (>0.5 ng/dL) and their viro-immunological status focusing our attention on mortality and length of hospital stay.

### 3.1. Patient Characteristics

Among the 289 HIV-positive patients evaluated, 74% were male, and the median age was 54 years [IQR: 43–62]. One hundred patients (35%) presented detectable viremia, and the median value of CD4+ T lymphocytes was 358 [IQR: 104–531]. Elevated PCT levels (0.5 ng/dL) were detected in 69 (23.8%) patients.

The median creatinine level for all patients was 0.92 mg/dL (IQR: 0.72–1.21). Patients with PCT < 0.5 ng/mL had a median creatinine level of 0.89 mg/dL (IQR: 0.7125–1.080), whereas those with PCT ≥ 0.5 ng/mL had a higher median creatinine level of 1.135 mg/dL (IQR: 0.8050–1.6775) (*p* < 0.001). The median NT-proBNP level for all patients was 362 pg/mL (IQR: 96–1607). Patients with PCT < 0.5 ng/mL had a significantly lower median NT-proBNP level of 133.5 pg/mL (IQR: 66.25–454) compared to those with PCT ≥ 0.5 ng/mL, who had a higher median NT-proBNP level of 1411 pg/mL (IQR: 592.75–4408.5) (*p* < 0.001). Platelets in the study population were 200.5 × 10^9^/L with a statistically lower value in the PCT ≥ 0.5 ng/mL group (*p* = 0.001). There was no statistically significant difference in the number of patients with detectable HIV-RNA between patients with PCT < 0.5 ng/mL and those with PCT ≥ 0.5 ng/mL (*p* = 0.981). The median CD4+ count for all patients was 358 cells/μL (IQR: 104–531). Patients with PCT < 0.5 ng/mL had a slightly higher median CD4+ count of 378 cells/μL (IQR: 104–572.25) compared to those with PCT ≥ 0.5 ng/mL, who had a lower median CD4+ count of 305 cells/μL (IQR: 91–511), although this difference was not statistically significant (*p* = 0.371). Seventeen subjects presented a QSOFA score ≥ 2 at the admission, not surprisingly, 12 were in the group with higher levels of PCT (<0.001). CRP levels in the general population were 51.0 mg/L [IQR: 13.25–143.9], with statistically significant higher levels in those who presented even PCT levels ≥ 0.5 ng/mL [163.4 mg/L (IQR: 62.8–204.7), *p* < 0.001]. The median length of hospital stay (LOS) for all patients was 14 days (IQR: 7.111–25.931). Patients with PCT < 0.5 ng/mL had an increased survival (*p* = 0.004) and a slightly shorter median LOS of 11.59 days (IQR: 6.577–24.555) compared to those with PCT ≥ 0.5 ng/mL, who had a median LOS of 16.0 days (IQR: 11.5–26.494) (*p* = 0.029). The main characteristics and comorbidities of the study population are summarized in Table 1.

### 3.2. Infective Diagnosis (Any Type of Infection)

Among the 289 patients included in this study, 72 patients did not present a confirmed infection and 217 patients had a diagnosis of infection as summarized in Table 2.

No statistical differences in age and Charlson score were found in the two groups. No differences were found even in the PCT levels in the two groups (*p* = 0.479). Patients with infection had a significantly higher median WBC count [7.69 × 10^3^/μL, (IQR: 5.3–12.2)] compared to those without infection [7.03 × 10^3^/μL, (IQR: 2.9–9.6)] (*p* = 0.011). Patients with a diagnosis of infection had a median CRP level of 52.5 mg/L [IQR: 14.7–140.0] compared to patients without infection [42.2 mg/L (IQR: 9.45–136.075)] (*p* = 0.381). The median CD4+ count for patients without infection was 344.5 cells/μL (IQR: 135–478), whereas for patients with infection, it was 358 cells/μL (IQR: 99–551) (*p* = 0.019). A total of 181 (83.4%) subjects with infection presented fever > 38 at the ED admission, compared to 52 in the other group (*p* = 0.031). Dyspnea and cough were the most frequent symptoms at the presentation at the ED (39.2% and 30.4%, respectively) in patients diagnosed with infection, with a significant difference in the two groups (*p* = 0.033 and *p* = 0.003, respectively). Indeed, pneumonia was the most frequent infection diagnosed in these patients (57.1%). Of these, we identified 27 (21%) with *P. jirovecii* pneumonia, 11 (8%) with tuberculosis, and 21 (17%) with COVID-19.

The Multivariate Cox regression analysis showed that once corrected for significant clinical, demographical, and comorbidity covariates, detectable levels of HIV-RNA [HR: 2.67 (IQR: 1.37–5.20), *p* = 0.004], WBC count [HR: 1.11 (IQR: 1.03–1.81), *p* = 0.003], and presentation with cough [HR: 2.40 (IQR: 1.12–5.16), *p* = 0.02] were independently associated to a high risk of having an infection in PLWH presented to the ED. As for the 72 cases without diagnosed infection, which presented fever, five were newly discovered HIV infections.

#### Bloodstream Infection

In the study population, 24 (8%) patients had a final diagnosis of BSI. The median age of these patients was 62 years (IQR: 55–66), significantly older than those who did not develop a BSI (*p* = 0.003). As a result, they also had a higher Charlson score due to the higher number of comorbidities (*p* = 0.003). Procalcitonin values in the BSI group were significantly higher [2.67 (IQR: 0.53–21.6)] than the other group (*p* < 0.001), as well as the PCR values [147 (IQR: 80–269) vs. 49 (IQR: 11–136)] (*p* < 0.001).

Most of the BSI group were primitive and probably due to prompt recognition in our ED, and in this group, three patients died during hospitalization. Not surprisingly, the BSI group had a longer hospitalization compared to those who did not have the BSI, with a statistically significant LOS of 19.5 days [IQR: 12.5–30.4] (*p* = 0.007). All the characteristics of the two groups are summarized in Table 3.

Once corrected for significant clinical, demographical, and comorbidity covariates, the Multivariate Cox regression analysis demonstrated that PCT > 0.5 ng/mL [HR: 8.05 (IQR: 2.54–25.46), *p* < 0.001] and older age [HR: 1.05 (IQR: 1–1.1), *p =* 0.02] were an independent risk factor for BSI in PLWH admitted to the ED.

### 3.3. Survival and Mortality

Non-survivors exhibited a significantly higher median procalcitonin (PCT) level (0.385 ng/mL, IQR: 0.098–2.34) compared to survivors (0.1 ng/mL, IQR: 0.05–0.345) (*p* = 0.002). Regarding cell counts, non-survivors had a significantly lower white blood cell (WBC) count (5.3 × 10^3^/μL, IQR: 4.55–8.4925) compared to survivors (7.84 × 10^3^/μL, IQR: 5.22–12.020) (*p* = 0.029), and a significantly lower platelet count (120.5 × 10^3^/μL, IQR: 72–197) compared to survivors (264 × 10^3^/μL, IQR: 150.25–271) (*p* = 0.001) as shown in Table 4.

Concerning the viro-immunological status, a statistically significant difference was observed in CD4+ counts between survivors (median: 369.5 cells/μL, IQR: 136.25–548) and non-survivors (median: 61 cells/μL, IQR: 14–186) (*p* < 0.001). At the same time, a detectable level of HIV viremia was found in 60% of deceased patients (*p* = 0.02).

Analyzing the main infectious syndromes of patients admitted to the emergency department (pneumonia, BSI, urinary tract infections), we found that mortality was significantly higher in those with pneumonia (survived 24% vs. deceased 45%; *p* = 0.02), we found no differences in BSI and urinary tract infections (UTIs). Finally, non-survivors had a significantly longer median length of hospital stay (17.972 days, IQR: 15.11–17.972) compared to survivors (13 days, IQR: 7–25) (*p* = 0.021).

Once adjusted for significant clinical, demographical, and comorbidity covariates, we identified that HIV-RNA detectable levels [HR: 3.66 (IQR: 1.41–9.48), *p* = 0.007] and PCT > 0.5 ng/mL [HR: 3.10 (IQR: 1.24–7.72), *p* = 0.015] were independent factors for in-hospital death in PLWH admitted to the ED.

In a sub-analysis, we also compared deaths in the pre-COVID-19 period with the pandemic period, without finding a statistically significant difference (*p* 0.17). We had 69 patients in the pre-COVID-19 period with three deaths (4.3%) and 220 patients in the COVID-19 period with 21 deaths (one with COVID-19) (9.5%).

### 3.4. Receiver Operating Characteristic (ROC) Curve Analysis of Procalcitonin (PCT) Value for Blood Stream Infection (BSI) and Mortality

**Figure 1:**
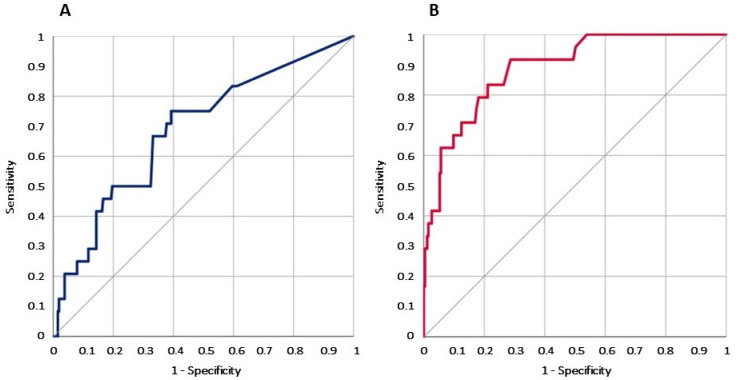
Graph of the ROC curve analysis for PCT values in PLWH admitted to the ED associated with mortality (**A**) and BSI (**B**).

The PCT value at admission in the ED had a good discrimination power for BSI and mortality. Not surprisingly, a direct correlation is stronger for BSI compared to mortality. In particular, the ROC AUC was 0.686 (0.573–0.800) for the PCT value in line with mortality. Conversely, the ROC AUC was 0.888 (0.826–0.950) for BSI (Figure 1).

## 4. Discussion

Although several studies have evaluated the prognostic and clinical relevance of procalcitonin for ED patients with fever [18,19], this represents the largest study conducted to date in the ED setting for PLWH.

The main finding of the present work is that for PLWH, the ED determination of PCT has a low discriminating value for an infective diagnosis. On the other hand, in these patients, the PCT value still has a prognostic value for overall mortality risk, and for the diagnosis of BSI (Figure 1).

The association of a high level of PCT with a poor prognosis has been widely analyzed in several clinical contexts [14,18,19,20,21]. Procalcitonin (PCT) has a proven clinical utility for risk stratification and antibiotic management, but its direct impact on patient outcomes remains controversial. Recent studies, particularly in intensive care units (ICUs), suggest through meta-analyses that the PCT-guided antibiotic treatment can improve survival and reduce the duration of therapy in septic patients [12,22]. However, the prognostic significance of PCT in patients admitted to the Emergency Department (ED) is less clear. Only a few studies conducted in this setting have shown a significant difference in PCT levels between surviving and non-surviving patients, indicating a potential predictive value that requires further validation [13,23,24,25]. Our study confirms these findings in PLWH, and a PCT > 0.5 ng/mL in the ED was associated with a higher in-hospital mortality risk in the study population.

As expected, we found that a lower CD4+ count in PLWH was associated with a higher mortality rate, as already shown in previous studies [26]. In contrast, a progressive increase in CD4+ count has been shown to be associated with increased survival [27]. Concerning the emergency setting, Huang et al. [28] analyzed the predictive value of CD4+ T lymphocyte counts in ICU patients. In their article, they found that lower levels were associated with 28-day mortality. Probably, the immunological status of patients with sepsis, not only those affected by HIV, remains a prognostic factor of mortality worthy of further investigation. Understandably, PLWH with unsuppressed viral loads, potentially related to consistently heightened inflammation [29,30], develop various AIDS and non-AIDS-related comorbidities that may cause disability and lead to death [31]. In our study, most of the patients with detectable HIV viremia were not on antiretroviral therapy because of a naïve condition or because of discontinuation.

In the PLWH study cohort, we also found an association between platelet count in the ED and overall mortality. This finding is well known, and, in particular, some research has shown how that a low value is linked in patients with sepsis to high mortality [32,33]. Regarding PLWH, Camon et al. showed in 160 patients with community pneumonia who required intensive care unit admission, that a low platelet value was a predictor of mortality on multivariate analysis [34].

In our cohort, pneumonia remains the main infectious syndrome of patients admitted to the Emergency Department, in particular, in those with poor prognosis. It has been already described that, as for the present population, pneumonia is the main reason to access the ED for PLWH [35,36] and could be the first presentation for a new HIV diagnosis [37].

Although many studies have been conducted on the use of procalcitonin in the emergency setting, providing guidance regarding special populations will provide future guidance to emergency physicians for proper diagnostic and prognostic framing. Indeed, this study lays the groundwork for subsequent investigations to implement the use of biomarkers in PLWH in the emergency setting to facilitate clinical management and will hopefully suggest insights for future investigations in this field.

### Study Limitations

Although the study was conducted in a large cohort of patients, some limitations remain. The retrospective and observational nature of the study limits the generalization of the results for every ED. Moreover, due to the design of the study conducted in the Emergency Department, we were not able to trace the nadir CD4+ values. Since this is a retrospective analysis, based on the evaluation of the ICD code at discharge, we could not differentiate between opportunistic and intercurrent infections. However, the main scope of our work was to evaluate the association between the procalcitonin value at the ED admission and its role as a prognostic marker in PLWH presented with fever in the ED setting. In addition, since the request of the procalcitonin was left at the ED physician’s discretion, we cannot exclude an indirect population selection. Finally, since no specific management was linked to the PCT results, we cannot evaluate the direct effect of PCT value on clinical management. In addition, the HIV test is not routinely performed in our ED for all patients with fever and indeed this may have led to a few missed HIV diagnoses; however, this limitation is mitigated by the fact that we have a 24/7 infectious disease consultant who coordinates antibiotic stewardship and helps the emergency medicine physicians to manage infective cases at the ED.

## 5. Conclusions

The clinical and diagnostic workup of patients with fever remains a challenge in the ED, and this is mostly true for PLWH. The present study confirms that raised procalcitonin levels in HIV patients admitted to the Emergency Department are associated with an increased risk of in-hospital mortality. Detectable viremia and lower CD4+ further amplifies this risk.

Pneumonia remains one of the main infectious syndromes related to febrile presentation at the ED for PLWH and needs to be promptly recognized and treated. However, the role of PCT in diagnosing an infectious origin of fever is very limited in this population.

On the other hand, our data confirm a good diagnostic reliability of PCT values for BSI diagnosis in HIV patients accessing the ED for fever.

These findings emphasize the potential utility of procalcitonin as a prognostic marker in HIV patients, aiding clinicians in identifying those at higher mortality risk, improving therapeutic strategies, and guiding appropriate surveillance. Further investigations are needed to confirm and expand these results.

## Figures and Tables

**Table 1:** Demographical and clinical characteristics of the patients included in the study. Values are shown separately according to the findings of elevated PCT values in the ED.

Variables	All Patients	PCT < 0.5	PCT > 0.5	*p* Value
	*n* = 289	*n* = 220	*n* = 69	
Age	54 [43–62]	50 [41–61]	59 [54–64]	<0.001
Charlson	7 [6–8]	6 [6–8]	7 [6–9]	0.006
Sex (M%)	214 (74)	163 (74)	51 (74)	0.977
Emergency Department Presentation (Laboratory tests)				
Creatinine (mg/dL)	0.92 [0.72–1.21]	0.89 [0.7125–1.08]	1.13 [0.80–1.67]	<0.001
Hb (g/dL)	12.6 [10.75–14.3]	13.05 [11.0–14.7]	11.5 [9.6–13.8]	0.002
Fibrinogen (mg/dL)	477 [364–663.5]	446.5 [344–616.75]	567 [463.5–811]	<0.001
WBC (×109/L)	7.6 [5.14–11.83]	7.30 [4.62–10.25]	9.18 [5.73–15.11]	0.002
Glucose (mg/dL)	105 [92.25–131.75]	103.5 [92–121]	121 [101.5–169.25]	<0.001
Procalcit. (ng/dL)	0.1 [0.05–0.45]	0.06 [0.05–0.13]	1.95 [0.92–4.515]	<0.001
Platelets (×109/L)	200.5 [141.0–270.7]	214 [152–285]	174 [119–211]	0.001
CRP (mg/dL)	51.0 [13.25–143.9]	34.4 [9.6–95.15]	163.4 [62.8–204.7]	<0.001
NT-proBNP (ng/L)	362 [96–1607]	133 [66.25–454]	1411 [592.75–4408.5]	<0.001
HIV detectable	100 (35)	76 (34)	24 (34)	0.971
CD4+ (cell/mm^3^)	358 [104–531]	378 [104–572.2]	305 [91–511]	0.371
Emergency Department Presentation (Symptoms)				
QSOFA 2+	17 (5.9)	5 (2.3)	12 (17.3)	<0.001
Fever > 38 °C	204 (70.6)	151 (68.6)	53 (76.8)	0.387
Dyspnea	104 (36)	72 (32.7)	32 (46.4)	0.029
Cough	76 (26.3)	65 (29.5)	11 (15.9)	0.016
Hemoptysis	4 (1.4)	4 (1.8)	0	0.334
Syncope	12 (4.2)	10 (4.5)	2 (3)	0.423
Diarrhea	17 (5.9)	17 (7.7)	0	0.008
Vomit	30 (10.4)	23 (10.5)	7 (10)	0.573
Abdominal pain	37 (12.8)	27 (12)	10 (14)	0.68
Chest pain	18 (6.2)	13 (6)	5 (7.2)	0.77
Comorbidities				
COPD	34 (11.8)	25 (11.4)	9 (13)	0.424
Hepatopathy	57 (19.7)	42 (19)	15 (22)	0.372
Cirrhosis	23 (8.0)	17 (7.7)	6 (8.7)	0.483
Diabetes	19 (6.6)	15 (7)	4 (6)	0.509
Heart failure	7 (2.4)	4 (2)	3 (4)	0.36
CKD	16 (5.5)	11 (5)	5 (7.2)	0.55
Infections				
BSI	24 (8.3)	5 (2.3)	19 (27.5)	<0.001
Pneumonia	124 (42.9)	99 (45)	25 (36.2)	0.21
Abdominal inf.	21 (7.3)	16 (7)	5 (7)	1
Urinary Tract inf.	21 (7.3)	16 (7)	5 (7)	1
Cutaneous inf.	6 (2)	6 (3)	-	0.35
Outcomes				
Death	24 (8.3)	12 (5.5)	12 (17.5)	0.004
LOS (days)	14 [7.11–25.9]	11.6 [6.57–24.55]	16 [11.5–13.8]	0.029

**Table 2:** Comparison of clinical and laboratory parameters between patients with and without infection.

Variables	No Infection *n* = 72	Infection *n* = 217	Univar. *p* Value	HR	Multiv. *p* Value
Age	56 [44–62]	53 [42–62]	0.41		
Charlson	7.5 [5.25–8.00]	6.0 [6.0–8.0]	0.3		
Emergency Department Presentation (Laboratory tests)					
PCT (ng/mL)	0.12 [0.05–0.28]	0.1 [0.05–0.49]	0.48	0.98 [0.45–2.11]	0.95
Creatinine (mg/dL)	0.96 [0.75–1.38]	0.91 [0.71–1.19]	0.12		
Hb (g/dL)	11.35 [9.6–13.6]	13.2 [11.1–14.8]	<0.001		
Fibrinogen (mg/dL)	468.0 [359.5–677.0]	477 [366.0–655.5]	0.72		
WBC (×109/L)	7.03 [2.91–9.60]	7.69 [5.3–12.2]	0.01	1.11 [1.03–1.18]	0.003
Glucose (mg/dL)	106 [92.5–137.25]	105 [92.25–129.75]	0.70		
Platelets (×109/L)	187 [115–251]	209.5 [150–278.5]	0.04		
CRP (mg/dL)	42.2 [8.32–147.25]	52.5 [14.7–140.0]	0.38	1.00 [0.98–1.00]	0.79
HIV detectable	17 (24)	83 (38)	0.31	2.67 [1.37–5.20]	0.004
CD4+ (cell/mm^3^)	344.5 [135.0–478.0]	358 [99–551]	0.02		
Emergency Department Presentation (Symptoms)					
QSOFA 2+	7 (9.7)	10 (4.6)	0.01		
Fever > 38 °C	52 (72.2)	181 (83.4)	0.03	0.62 [0.31–1.23]	0.173
Dispnea	19 (26.4)	85 (39.2)	0.03		
Cough	10 (13.9)	66 (30.4)	0.003	2.40 [1.12–5.16]	0.024
Hemoptysis	0 (0)	4 (1.8)	0.32		
Syncope	6 (8.3)	6 (2.8)	0.05		
Diarrhea	5 (6.9)	12 (5.5)	0.42		
Vomit	8 (11.1)	22 (10.1)	0.48		
Abdominal pain	11 (15.3)	26 (12)	0.54		
Chest pain	7 (9.7)	11 (5.1)	0.54		
Comorbidities					
COPD	2 (2.8)	32 (14.7)	0.003		
Hepatopathy	21 (29.2)	36 (16.6)	0.02		
Cirrhosis	6 (8.3)	17 (7.8)	0.53		
Diabetes	4 (5.6)	15 (6.9)	0.46		
Heart failure	2 (2.8)	5 (2.3)	0.99		
CKD	7 (9.7)	9 (4.1)	0.08		
Infections					
BSI	-	24 (11)			
Pneumonia	-	124 (57.1)			
Abdominal inf.	-	21 (9.7)			
Urinary Tract inf.	-	21 (9.7)			
Cutaneous inf.	-	6 (2.8)			
Outcomes					
Death	7 (9.7)	17 (7.8)	0.62		
LOS (days)	9.76 [6.5–17]	15.4 [8.0–27.5]	0.001		

**Table 3:** Comparison of clinical and laboratory parameters between patients with and without a blood stream infection.

Variables	BSI *n* = 24	No BSI *n* = 265	Univar. *p* Value	HR	Multiv. *p* Value
Age	62 [55–66]	53 [42–61]	0.003	1.05 [1–1.1]	0.02
Charlson	8 [6.25–11.75]	6 [6–8]	0.003		
Emergency Department Presentation (Laboratory tests)					
PCT (ng/mL)	2.67 [0.53–21.6]	0.09 [0.05–0.28]	<0.001	8.05 [2.54–25.46]	<0.001
Creatinine (mg/dL)	1.22 [1.06–3.86]	0.91 [0.71–1.14]	<0.001		
Hb (g/dL)	10.95 [9.2–14.0]	12.9 [10.8–14.3]	0.035		
Fibrinogen (mg/dL)	529.5 [424–662]	471 [360–663]	0.172		
WBC (×109/L)	8.74 [6.4–16.8]	7.48 [4.9–11.5]	0.058	1.06 [ 0.99–1.13]	0.12
Glucose (mg/dL)	123 [93.0–173]	104 [92–129]	0.072		
Platelets (×109/L)	142 [120–198]	208 [150–273]	0.011		
CRP (mg/L)	147 [80–269]	49 [11–136]	<0.001	1.00 [0.99–1.01]	0.34
HIV detectable	10 (41.7)	90 (34)	0.503	1.88 [ 0.62–5.73]	0.27
CD4+ (cell/mm^3^)	284 [69–503]	360 [104–548]	0.219		
Emergency Department Presentation (Symptoms)					
QSOFA 2+	2 (8.3)	15 (5.7)	0.422		
Fever > 38 °C	21 (87.5)	212 (80.0)	0.278	0.45 [0.11–1.83]	0.26
Dispnea	8 (33.3)	96 (36.2)	0.483		
Cough	2 (8.3)	74 (27.9)	0.025	0.28 [0.05–1.4]	0.28
Hemoptysis	0	4 (1.5)	0.706		
Syncope	0	12 (4.5)	0.346		
Diarrhea	0	17 (6.4)	0.219		
Vomit	2 (8.3)	28 (10.6)	0.535		
Abdominal pain	2 (8.3)	35 (13.2)	0.75		
Chest pain	1 (4.2)	18 (6.8)	0.38		
Comorbidities					
COPD	6 (25.0)	28 (10.6)	0.047		
Hepatopathy	5 (20.8)	52 (19.6)	0.531		
Cirrhosis	4 (16.7)	19 (7.2)	0.110		
Diabetes	3 (12.5)	16 (6.0)	0.201		
Heart failure	2 (8.3)	5 (1.9)	0.10		
CKD	3 (12.5)	13 (4.9)	0.13		
Infections					
BSI (primitive)	20 (83)	-	<0.001		
Pneumonia	-	124 (46.8)	<0.001		
Abdominal inf.	1 (4.2)	20 (7.5)	1		
Urinary Tract inf.	3 (12.5)	18 (6.8)	0.4		
Cutaneous inf.	-	6 (2.3)	1		
Outcomes					
Death	3 (12.5)	21 (7.9)	0.43		
LOS (days)	19.5 [12.5–30.4]	13 [7.0–25]	0.007		

**Table 4:** Factors associated with death in PLWH with fever admitted to the Emergency Department.

Variables	Survivors *n* = 265	Non-Survivors *n* = 24	Univariate *p* Value	Hazard Ratio	Multiv. *p* Value
Age	53 [42–62]	59 [45.2–62.0]	0.300	1.01 [0.97–1.05]	0.66
Charlson	7 [6–8]	8 [6–10]	0.023	1.14 [0.95–1.36]	0.17
Emergency Department Presentation (Laboratory tests)					
PCT (ng/mL)	0.1 [0.05–0.345]	0.385 [0.098–2.34]	0.002	3.1 [1.25–7.72]	0.015
Creatinine (mg/dL)	0.93 [0.75–1.2175]	0.68 [0.5525–1.220]	0.013		
Hb (g/dL)	12.7 [10.8–14.3]	12.3 [10.2–13.8]	0.39		
Fibrinogen (mg/dL)	477 [374–673]	448 [272–567]	0.03		
WBC (×109/L)	7.840 [5.22–12.0]	5.3 [4.55–8.49]	0.03		
Glucose (mg/dL)	104 [92–128]	113 [103–154]	0.07		
Platelets (×109/L)	208.5 [150.25–271]	120.5 [72–197]	0.001		
PCR (mg/dL)	50.9 [13.25–143.05]	67.9 [14.6–194.6]	0.50		
CD4+ (cell/mm^3^)	369.5 [136.25–548.00]	61 [14–186]	<0.001		
HIV detectable	85 (32)	15 (60)	0.02	3.66 [1.41–9.48]	0.007
QSOFA 2+	14 (5.3)	3 (12.5)	0.16		
Emergency Department Presentation (Symptoms)					
Fever > 38 °C	217 (81.9)	16 (66.7)	0.07		
Dispnea	93 (35.1)	11 (45.8)	0.20		
Cough	73 (27.5)	3 (12.5)	0.08		
Hemoptysis	4 (1.5)	0 (0)	0.71		
Syncope	12 (4.5)	0 (0)	0.35		
Diarrhea	17 (6.4)	0 (0)	0.22		
Vomit	30 (11.3)	0 (0)	0.06		
Abdominal pain	36 (13.6)	1 (4.2)	0.33		
Chest pain	18 (6.8)	0 (0)	0.38		
Comorbidities					
COPD	32 (12.1)	2 (8.3)	0.44		
Hepatopathy	54 (20.4)	3 (12.5)	0.26		
Cirrhosis	21 (7.9)	2 (8.3)	0.59		
Diabetes	15 (5.7)	4 (16.7)	0.06		
Heart failure	5 (2)	2 (8.3)	0.11		
CKD	13 (5)	3 (12.5)	0.14		
Infections					
BSI	21 (7.9)	3 (12)	0.43		
Pneumonia	64 (24)	11 (45)	0.02		
Abdominal inf.	21 (8)	0 (0)	0.23		
Urinary Tract inf.	21 (8)	0 (0)	0.23		
Cutaneous inf.	6 (2.3)	0 (0)	1		
Outcomes					
LOS (days)	13 [7–25]	18 [15–29]	0.021		

## Data Availability

The data presented in this study are available on reasonable request from the corresponding author.
